# Performance of Foundry Sand Concrete under Ambient and Elevated Temperatures

**DOI:** 10.3390/ma12162645

**Published:** 2019-08-20

**Authors:** Hazrat Bilal, Muhammad Yaqub, Sardar Kashif Ur Rehman, Muhammad Abid, Rayed Alyousef, Hisham Alabduljabbar, Fahid Aslam

**Affiliations:** 1 Department of Civil Engineering, City University of Science & Information Technology, Peshawar 25000, Pakistan; 2 Department of Civil Engineering, University of Engineering & Technology, Taxila 47050, Pakistan; 3 Department of Civil Engineering, COMSATS University Islamabad, Abbottabad Campus, Abbottabad 22060, Pakistan; 4 College of Aerospace and Civil Engineering, Harbin Engineering University, Harbin 150090, China; 5 Department of Civil Engineering, College of Engineering in Al-Kharj, Prince Sattam bin Abdulaziz University, Al-Kharj 11942, Saudi Arabia

**Keywords:** foundry sand concrete, strength properties, ultrasonic tests, elevated temperatures, residual compressive strength, explosive spalling

## Abstract

Waste foundry sand (WFS) is the by-product of the foundry industry. Utilizing it in the construction industry will protect the environment and its natural resources, and enable sustainable construction. WFS was employed in this research as a fractional substitution of natural sand by 0%, 10%, 20%, 30%, and 40% in concrete. Several tests, including workability, compressive strength (CS), splitting tensile strength (STS), and flexural strength (FS), ultrasonic pulse velocity (USPV), Schmidt rebound hammer number (RHN), and residual compressive strengths (RCS) tests were performed to understand the behavior of concrete before and after exposure to elevated temperatures. Test findings showed that the strength characteristics were increased by including WFS at all the phases. For a substitute rate of 30%, the maximum compressive, splitting tensile, and flexural strength were observed. Replacement with WFS enhanced the 28-day compressive, splitting tensile, and flexural strength by 7.82%, 9.87%, and 10.35%, respectively at a 30% replacement level, and showed continuous improvement until the age of 91 days. The RCS of foundry sand concrete after one month of air cooling at ambient temperature after exposing to 300 °C, 400 °C, 500 °C, 600 °C, 700 °C, and 800 °C was found to be in the range of 67.50% to 71.00%, 57.50% to 61.50%, 49.00% to 51.50%, 38% to 41%, 31% to 35% and 26% to 31.5% of unheated compressive strength values for 0% to 40% replacement of WFS, respectively. The RCS decreases with increasing temperature; however, with increasing WFS, the RCS was enhanced in comparison to the control samples. In addition, the replacement of 30% yielded excellent outcomes. Hence, this study provides a sustainable construction material that will preserve the Earth’s natural resources and provide a best use of WFS.

## 1. Introduction

The construction industry plays a vital role in the economic development of any country. Concrete can be classified as the most abundantly used material of the construction industry. The only material used more than concrete by man is water [1]. Currently, concrete is a ubiquitously used construction material (20–35 billion tons annually usage) in the world. Its demand is increasing gradually because of its numerous useful properties, such as excellent resistance to water, its ability to mold into various forms and sizes, affordability, high strength, and durability. Moreover, fire resistance is the single most unique yet distantly observed aspect of concrete [1,2,3]. Raw materials for concrete are obtained from natural resources which are depleting at a fast rate and also lead to the degradation of the natural environment. As the construction industry consumes a large amount of naturally available material, sustainable development in the construction industry is quite important [4,5]. Thus, the alternative is the utilization of industrial by-products and solid wastes such as waste foundry sand, bottom ash, slag, silica fume, and fly ash in producing concrete. The utilization of industrial by-products or waste materials in concrete compensates for the lack of natural resources, solving the disposal problems and creating a way to find the alternative technique to save natural resources.

Foundry sand is a molding sand that is used by the foundry industry owing to its easy accessibility, low cost, heat damage resistance, and bonding with other binders and organic materials. This sand is quite higher in quality than natural sand. Foundry industries utilize this sand time and again. When this sand remains no longer able to be used further in foundry industries, then it is removed and termed as waste foundry sand (WFS). Waste foundry sand is also referred to as used foundry sand (UFS) or spent foundry sand (SFS) [6]. Fine particles in UFS are good enough. The type of metal that is to be poured is responsible for its physical and chemical properties, and even these properties can be changed from one foundry to another with a slight difference. There are two types of foundry sand: One is named as Clay-bonded (Green sand) and the other one is named as chemically bonded sand. Metal casting foundries in the U.S. dispose of about nine million tons of spent sand in landfills per year [7]. According to the industry estimates, almost 100 × 10^6^ tons of foundry sand is used in production in a year; out of this amount, about four to seven million tons are wasted per annum and offered for recycled use [8,9].

Numerous researchers investigated the use of used foundry sand in various civil engineering applications such as highway applications [10], controlled low-strength material [11], and in hot mix asphalt concrete [12]. Numerous researchers have testified the usage of WFS in concrete as a fractional substitution of fine aggregates [13,14,15,16,17,18,19].

Siddique et al. [13,14] evaluated the effective use of WFS. In this research study, normal sand was replaced with WFS by 10% to 30% at an increment of 10%. Tests of compressive, splitting tensile, and flexural strengths were performed after 28 and 56 days [13]. Similarly, the compressive, splitting tensile, flexural strength, and modulus of elasticity tests were determined after 28, 56, 91, and 365 days [14]. The incorporation of WFS as a fractional substitution up to 30 led to an increase in compressive strength (CS), splitting tensile strength (STS), the modulus of elasticity, and flexural strength (FS). The optimum substitution level of fine aggregates with WFS was found to be 30%. Siddique et al. [15] examined the effect of WFS on concrete compressive strength and splitting tensile strengths at 28, 90, and 365 days of age. The proportion of fine aggregate replaced ranged from 0% to 60% at the increment of 10%. Cubes of 150 mm and cylindrical specimens of 150 × 300 mm sizes were used to find the compressive strength and splitting tensile strengths at all ages, respectively. A 30% substitution of fine aggregate with WFS was found to be optimum, with a precaution that general replacement should not exceed a 50% replacement level. The incremental trend in compressive and splitting tensile strengths were observed with the age also. Increases of 24.03%, 19.23%, and 14.23% in splitting tensile strengths for 30%, 40%, and 50% respectively were observed, while 4.62% and 2.39% increments were observed in compressive strength for 30% and 40% replacement levels, respectively, at the age of 28 days. Increases of 25.18%, 22.55%, and 19.92% in splitting tensile strengths for 30%, 40%, and 50%, respectively were observed at the age of 90 days. Siddique et al. [16] assessed the properties of two different (M20 and M30) grades of concrete in which WFS was partially replaced with natural sand by (0–20%), with an incremental interval of 5%.

In the previous study, Khatib et al. [20] substituted WFS in various quantities for fine aggregate in plain concrete—i.e., from 0% to 100% by an incremental interval of 20%—and witnessed a continuous slump loss, as well as a loss of mechanical properties. Based on the type of metal, binder, and combustible used, the chemical composition may vary up to some extent, and its efficiency may also be affected. Salokhe et al. [21] found that in case of strength gain, concrete produced with WFS from ferrous foundries performs better than concrete with nonferrous WFS. The water absorption capability depends on the carbon content; more water with high carbon content will be absorbed. Similarly, different authors have reported its behavior in concrete with different results. Siddique et al. [22] determined that similar to plain concrete, the compressive strength of self-compacting concrete also improved by replacing WFS with normal sand by up to 15%. Pathariya et al. [23] reported that a mixture of 60% WFS showed maximum strength. Siddique et al. [15] stated that at 28 days, 56 days, and 365 days of curing, the strengths of concrete mixes with 30%, 40%, and 50% WFS exceeds that of the control mix. Etxeberria et al. [24] examined concrete made with two qualities of WFS that were named green and chemical foundry sand and evaluated that both concrete samples possess more satisfactory strength than reference concrete at a high water to cement (w/c) ratio. Incremental trend in strength of concrete with the inclusion of WFS was observed by several researchers, and the author’s current study is in line with these researchers [13,14,15,17,25,26,27].

Compressive, splitting tensile strengths and flexural strengths tests were measured at the ages of 7, 28, 56, 91, and 365 days, and noticed an incremental trend of up to 15% replacement level for mutual (M20 and M30) grades of concrete. Ultrasonic pulse velocity (USPV) tests were evaluated at 28, 91, and 365 days of age. Ultrasonic pulse velocity (USPV) values also exhibited increment in values, with an increment in replacement level up to 15%. Singh and Siddique et al. [17,18] evaluated the strength and durability aspects of concrete prepared with a partial replacement of natural sand with spent foundry sand with 0–20% dosage, with an incremental interval of 5%. Strength aspects, i.e., the compressive strength, splitting tensile strength, and modulus of elasticity tests and durability aspects i.e., ultrasonic pulse velocity tests, rapid chloride permeability tests, and abrasion resistance tests were carried out. Compressive strengths, splitting tensile strengths, and the modulus of elasticity tests were performed at the age of 7, 28, and 91 days. An ultrasonic pulse velocity test was performed at 28 days and 91 days of age. Results showed a slight rise in concrete strength and durability by adding WFS as a fractional substitute of sand up to a substitutional level of 15%. Dash Kumar et al. [19] reported the effective utilization of waste foundry sand in concrete, and concluded that WFS can be effectively utilized as a substitution of fine aggregate up to 20% without compromising the mechanical and physical properties. The inclusion of waste foundry sand increase the value for ultrasonic pulse velocity, which is an indication of good density, homogeneity, and uniformity of concrete.

The residual strength of hardened concrete after fire when it cools down changes depending upon the maximum temperature attained, mix proportion, aggregate type, fire exposure intensity, and duration of fire [28,29,30]. Greater reduction would be occurred in the residual compressive strength at the higher rate of cooling [29]. Lee et al. [30] observed a rapid drop in compressive strength at temperature level above 400 °C. Residual compressive strength at 600 °C and 800 °C was 57% and 18% of the unheated samples, respectively. The failure at higher temperature is because of the development of cracks, variations in the chemistry, and an increase in internal pore pressure as a result of the evaporation of water [31,32]. Arioz et al. [33] reported that the compressive strength, ultrasonic pulse velocity, and rebound number decrease with any increase in temperature. A slight reduction was observed until a temperature of 400 °C, and sharp reduction was observed beyond 400 °C. The longer the exposure duration to elevated temperatures, the larger the fall in compressive strength due to the crack’s development and material decay. Most of the strength drop-down happens within the period of the first 30 days of long-term exposure [34].

Chang et al. [35] used concrete cylindrical specimens of size 150 × 300 mm to observe the influence of elevated temperatures on compressive strength. Cylindrical specimens were exposed to temperature levels in the range of 200 to 800 °C with an increment of 200 °C. The residual compressive strength at 200, 400, 600, and 800 °C was 90%, 65%, 40%, and 15% of the unheated samples, correspondingly. Hager [36] found that a decrease in strength of concrete starts at 300 °C. Beyond 400 °C, the strength decreases more rapidly due to the degradation of calcium silicate hydrate (CSH) gel. After 900 °C, CSH breaks down completely, so the temperature range of 400 to 900 °C is the critical temperature range for concrete compressive strength.

The already published literature such as Bhardwaj et al. [37], Bradshaw et al. [38], and Mavroulidou et al. [39] and other researchers such as [13,14,15,17,25,26,27] performed many tests on concrete comprising WFS as a partial replacement of sand at ambient temperatures. Since the elevated temperature is a catastrophic phenomenon, the behavior of WFS concrete should be evaluated under elevated temperatures. So, the published literature lacks studies related to effect of elevated temperatures on foundry sand concrete, regarding its resistance to fire in terms of spalling and residual compressive strength after exposure to fire/elevated temperatures. According to the author’s knowledge, the behavior of WFS concrete after exposure to elevated temperature has rarely been evaluated. The main objective of this research work is to explore the behavior of WFS concrete under elevated temperatures and compare the behavior of WFS concrete under elevated temperatures and ambient temperatures. Residual compressive strength after exposure to elevated temperature and resistance against fire in terms of a spalling phenomenon has been carefully evaluated.

## 2. Research Material

### Materials and Mix Proportions

**Figure 1:**
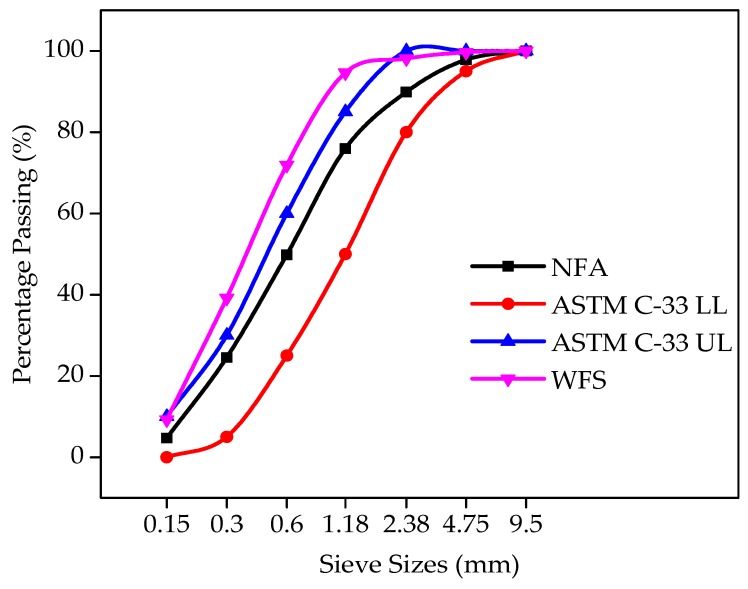
Sieve analysis results of natural fine aggregate (NFA) and waste foundry sand (WFS) in line with ASTM C-33 limits.

**Figure 2:**
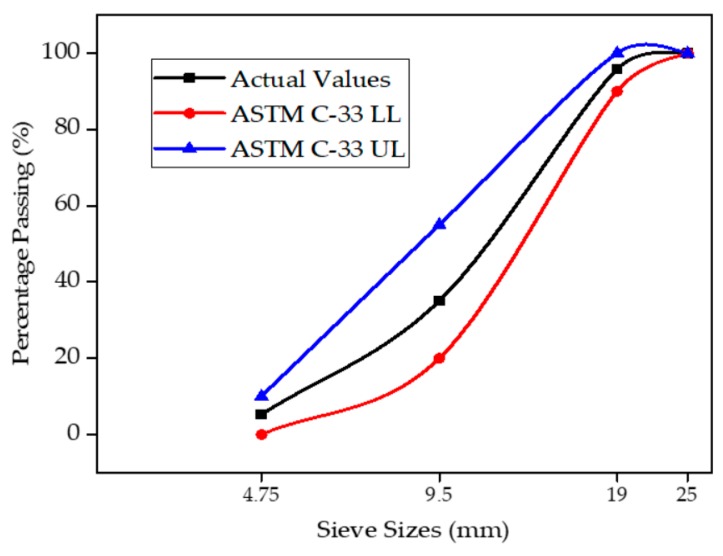
Particle size distribution curve of coarse aggregate in line with ASTM C-33 limits.

Ordinary Portland cement (OPC, Type I) was provided by the Fauji cement company in Pakistan with the chemical composition and physical properties complying with the standard specification of ASTM C-150 [40]. The chemical composition of OPC used in mix is given in Table 1, while the local available sand of Lawrencepur with a nominal size of 4.75 mm was used as fine aggregate. Local available Margalla crushed angular aggregate of 19-mm size was used as a nominal maximum size of coarse aggregate. The properties of fine and coarse aggregates were found to confirm the requirements of ASTM C-33 [41]. Locally available WFS was used as a fractional substitution of fine aggregates. Foundry sand fineness modulus and bulk density was observed to be lower than that of natural sand. The chemical properties of foundry sand are shown in Table 2. Sieve analysis and the physical properties of aggregates are shown in Figure 1 and Figure 2, and Table 3.

Sieve analysis of WFS and natural fine aggregate (NFA) was carried out to observe the particle size distribution pattern of WFS and NFA. The sieve analysis evaluation of WFS and normal sand showed that the WFS is a finer material than NFA. The sieve analysis pattern of WFS and NFA is presented in Figure 1.

Sieve analysis of coarse aggregate was carried out to observe the particle size distribution pattern of coarse aggregate. The sieve analysis pattern of coarse aggregate is presented in Figure 2. The particle size distribution curve of actual used coarse aggregate is within the limits of ASTM C-33 limits.

Five mixtures incorporating WFS as a fractional substitution of natural sand were prepared. Normal sand was substituted by WFS by weight. The proportion of replacement ranged from 0% to 40% at the accretion of 10%. The mix proportions to be used are provided in Table 4. Three specimens were cast for each replacement level and for each day of testing as well. Similarly, three specimens were cast for each replacement level, and for each level, the temperature ranged from 300 °C to 1000 °C at the incremental interval of 100 °C. Mix concrete without WFS was referred to as control mix and designed as per ACI-211.1-91 [42] and ACI 318-08 [43]. Mixture machine mixing was done for all the concrete mixes. Trial mixes were prepared to finalize the control or reference mix design with no addition of WFS. The target compressive strength set for control or referenced concrete mix at 28 days of age was 28 MPa.

## 3. Experimental Program

### 3.1. Fresh State Testing Procedure

Wet/fresh state characteristics of concrete such as slump and compacting factor tests have been idenfied according to ASTM C143/143M [44] and BS 1881-103 [45] standards, respectively. For compressive and splitting tensile strengths, specimens of 150 × 300 mm (6 × 12 inches) concrete cylinders were casted, while prism beams of 150 × 150 × 500 mm (6 × 6 × 20 inches) were casted for finding the flexural strengths. The compressive strengths, splitting tensile strengths, and flexural strengths were performed at the age of 7 days, 28 days, 56 days, and 91 days of standard curing as per ASTM C192/C192M [46].

The mean net value of three specimens was used for all the calculations at all the ages and replacement levels. All the samples were casted at ambient temperature. Casted samples were protected with plastic sheets and left in a molding yard for 24 h at ambient temperature, and then demolded and kept in water for the required age of curing and test. Compressive strengths, splitting tensile, and flexural strengths tests were performed as per ASTM standards C39/C39M [47], C496/C496M [48], and C293 [49], respectively.

### 3.2. Ultrasonic Pulse Velocity (USPV) Test

The USPV test mostly involves measuring the electronic wave velocity passing through the concrete specimen, which is used to diagnose the quality of concrete. A USPV test for concrete incorporating WFS as a partial substitute of sand was measured in the way discussed bleow.

(i) After the completion of standard curing (28 days) the samples were carried out from the curing tank and then dried at ambient temperature for seven days.
(ii) The USPV test was conducted at the seventh day after the completion of standard curing.
(iii) After that, the samples were then exposed to elevated temperatures of 300, 400, 500, 600, 700, 800, 900, and 1000 °C for one-hour at peak temperature level.
(iv) After being exposed to elevated temperatures, the samples were placed in surrounding air/open to sky environment for 30 days.
(v) A USPV test was conducted again at the 30th day after being exposed to the surrounding air/open to sky environment.

The ultrasonic pulse velocity of the concrete samples were measured by implementing the technique specified in BS EN 12504-4:2004 [50]. USPV tests were performed with the help of Portable Ultrasonic Non-Destructive Digital Indicating Tester (PUNDIT) on the post-heated/fired and unfired/unheated concrete cylinders. The position for measuring the pulse velocity values remained the same after exposure to the specified temperature as before heating. Petroleum jelly was used to make the surface of concrete cylinders smooth. The transmitting and receiving transducers were positioned on the opposite faces of the concrete cylinders. Four readings were taken at each sample, and the average value was recorded.

### 3.3. Schmidt Rebound Hammer Number (RHN) Test

The test of the rebound hammer number (RHN) is appropriate for both the laboratory and the field. The surface smoothness influences the rebound number. An RHN test for concrete incorporating WFS as a partial substitute of sand was measured in the way discussed bleow.

(i) After the completion of standard curing (28 days), the samples were carried out from the curing tank and then dried at ambient temperature (25 °C) for seven days.
(ii) The RHN test was conducted on the seventh day after completion of standard curing.
(iii) After that, the samples were then exposed to elevated temperatures of 300, 400, 500, 600, 700, 800, 900, and 1000 °C for 1 h at a peak temperature level.
(iv) After being exposed to elevated temperatures, the samples were placed in the surrouding air/open-sky environment for 30 days.
(v) An RHN test was conducted again at the 30th day after being exposed to the surrounding air/open-sky environment.

The rebound hammer test was performed by implementing the techniques specified in BS EN 12504-4:2004 [50], on the unfired/unheated and post-heated/fired concrete cylinders. Six readings were taken at each sample, and the average value was recorded. The rebound hammer tests belongs to the catogory of surface hardness surveys [51].

### 3.4. Heating Procedure/Fire Exposure

**Figure 3:**
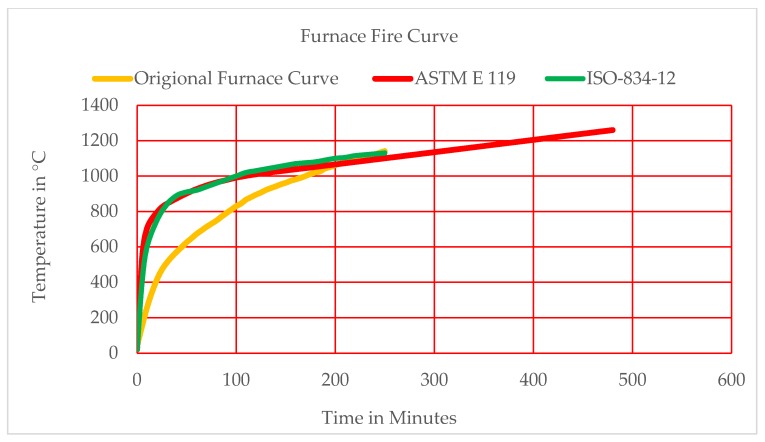
Used furnace temperature variation times in this present study.

After completing the period of 28 days of standard curing/watering, the entirely cured samples were picked out from the curing tank and dried out at ambient temperature for seven days. The concrete specimens (cylinders of 150 × 300 mm size) were fired/heated in an electric furnace for the temperature levels of 300, 400, 500, 600, 700, 800, 900, and 1000 °C at a mean rate of 4.5 °C/min. The level of temperature/hotness was increased in accordance with the fire curve of ISO-834-12 [52]. The highest/peak temperature was kept for a duration/period of one hour, and then samples were allowed to cool down slowly to room temperature overnight in the closed furnace. Once cooled, the specimens were taken out of the furnace and stored in an open air environment for 30 days. The time–temperature curve is illustrated in Figure 3. The temperature graph revealed a similar trend to those of ISO-834-12 and ASTM E119, respectively [52,53]. The samples were tested for residual compressive strength after the completion of 30 days of cooling in the surrounding air after exposure to different temperature levels ranging from 300 to 1000 °C at the increment of 100 °C. Then, the average residual strength values were recorded for all the specimens at each temperature. To study the behaviour of Foundry sand concrete exposed to elevated temperatures, three specimens were used for each of the following temperatures: 300, 400, 500, 600, 700, 800, 900, and 1000 °C. It is important to mention here that the elevated temperature response was noted only for specimens that were cured for 28 days.

## 4. Results and Discussion

### 4.1. Fresh State Testing Results

Wet concrete workability is the combination of composite properties that contains the necessities of ease in compaction, mobility, finishibility, and placeability. Slump is the indicative measurement of consistency or workability of concrete. The compacting factor is also in correspondance with the slump values. The slump value was noted to decrease with the incorporation of WFS because of the finer particles present in foundry sand. Water demand escalated with the escalating percentage substitution of foundry sand. Beyond 30% replacement, water demand increased in order to achieve the desired workability. As observed, the slump values decreased with the increase in replacement level. This might be due to the void-filling act of the WFS, as its particles are finer than the natural sand, which contributes high unity to the mix. The mix comprised of a high level of substitution of waste foundry sand content tends to become harsh, sticky, and stiff/inflexible beyond 30% replacement. Up to 30%, the mix was not so harsh, as we observed in mixing and placing. At a replacement rate of 30%, nearly a 15% decrease in slump value was noted. At the replacement point of 40%, the decrease in slump value was boosted up to around 31%. This reduction in workability is likely owing to the existence in WFS of water absorbing finer particles, i.e. clay-type fine material, ashes, and impurities, etc., which are accountable for reducing the fluidity of wet concrete and escalating the water demand. Similarly, a reducing trend in compacting factor (CF) values has also been noted with the increasing WFS level. The findings of this research are in agreement with those observed by [13,14,24,54,55,56]. Table 5 shows the results of the fresh properties of all the mixtures.

### 4.2. Compressive Strength Results

The compressive strength (CS) for all the mixtures consisting of WFS as a partial substitute of sand i.e., 0–40% at the increment of 10% was performed at the age of 7, 28, 56, and 91 days. The CS increased by up to 30% substitution level in linear behavior, and at the substitution level of 40%, the strength is nearly equal to the strength of the control mixture at all the ages. There is an increase in compressive strength at each replacement level, and a maximum increase in compressive strength was observed at a 30% replacement level. At 7 days of age, the percentage increase in CS as compared to the control mixture was 2.53%, 4.56%, 7.62%, and 1.45% for mixtures comprising 10%, 20%, 30%, and 40%, respectively. The concrete mixture containing 30% WFS at 7 days of age had a maximum compressive strength of 23.30 MPa, i.e., 7.62% higher than that of the control concrete. Concrete mixtures comprised of WFS of up to 10%, 20%, 30%, and 40% at 28 days of age gained 2.67%, 4.72%, 7.82%, and 1.65%, respectively, higher CS than that of the control mix.

**Figure 4:**
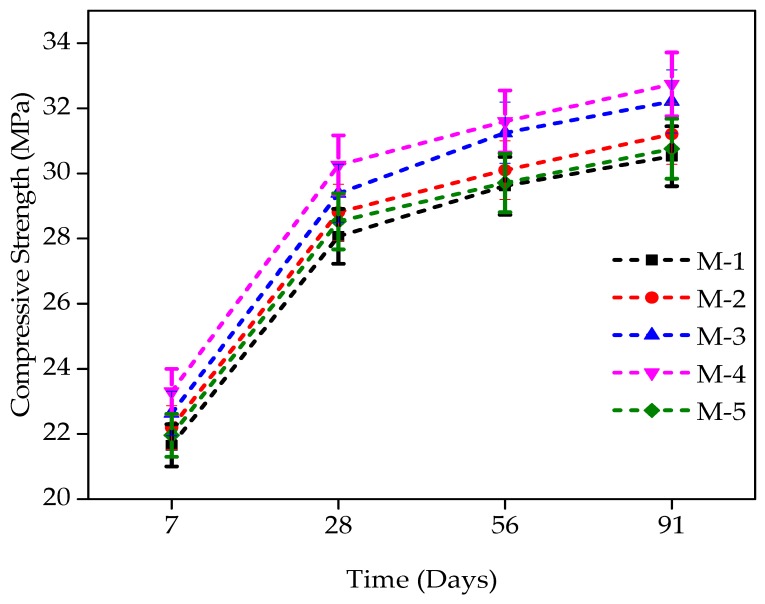
Compressive strength development of WFS concrete at various ages [47].

At 56 days of age, the enhancement in CS of concrete comprising of 10%, 20%, 30%, and 40% WFS respectively over that of control mixture was 7.13%, 11.31%, 12.59%, and 5.86%, respectively. Similarly, at the level of 91 days, there were 11.18%, 14.78%, 16.65%, and 9.59% increases in CS for concrete mixtures comprising of 10%, 20%, 30%, and 40% WFS respectively over that of control concrete. From Figure 4 mentioned below, the variation in CS enhancement can also be easily observed. It was noted that at all ages, concrete mixture comprising of 30% WFS as a partial sand substitute exhibited a higher CS value than that of the control concrete mixture. It was also noted that the strength-increasing pattern of concrete comprising WFS as a fractional substitute of sand with the age was similar to that of control concrete.

This is may be due to the existence in WFS of more fine particles that acted as excellent packing material and eventually resulted in a denser concrete mix [57]. The void-filling of finer particles decreases the pores among the concrete component, and results in a dense matrix. A reduction in the electrical conductance of concrete has also occurred [17]. The presence of silica content would have helped in the formation of CSH gel. It is undoubtedly because of the packing behavior of the matrix particles [13,14,15]. The strength reduction was noted beyond 30% replacement. This significant drop in CS with the incoporation of 40% WFS could possibly be due to an increase in the surface area of fine particles leading to a reduction of the water–cement gel in matrix; hence, the binding process of coarse and fine aggregate is not carried out correctly [17]. The findings of this research in terms of CS are in line with several others research findings [13,14,15,17,25,26,27].

The findings of distinct mixtures for compressive strength (CS) incorporating WFS at various ages are shown in Figure 4. Concrete mixtures produced with WFS could be noted as having greater CS than control concrete. The control mixture CS was around 28.1 MPa after 28 days of curing, and its value is very close to the mixture containing 40% WFS. The maximum strength was observed at a 30% replacement level, as clear from Figure 4.

### 4.3. Splitting Tensile Strength Results

**Figure 5:**
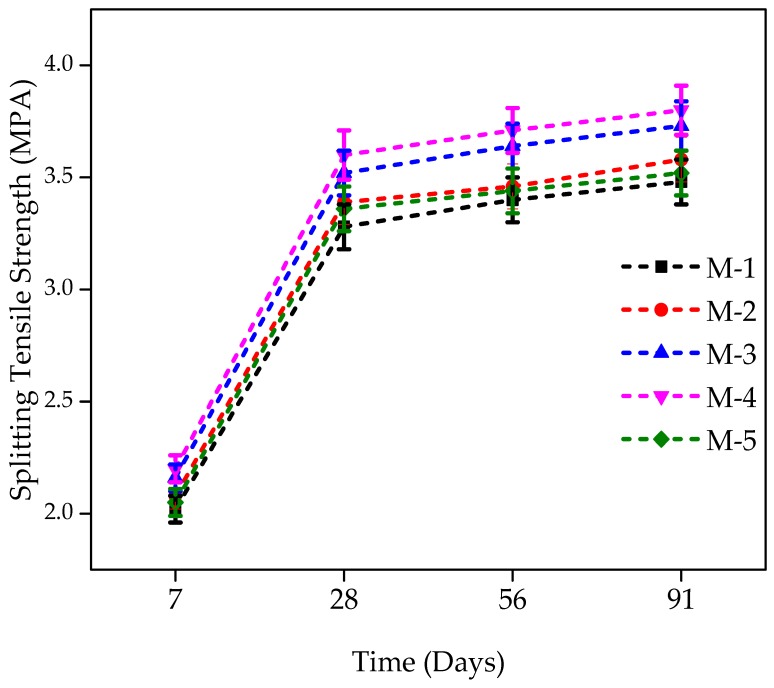
Splitting tensile strength development of WFS concrete at various ages [48].

The splitting tensile strength (STS) for all the mixtures consisting of WFS as a partial substitute were performed at the age of 7, 28, 56, and 91 days. The STS behavior of the control and WFS concrete mixtures are shown in Figure 5. The strength gain trend of WFS-based concrete mixtures is compatible with the trend toward compressive strength (CS). With the increased content of WFS up to a replacement level of 30%, an increase in STS was noted. The STS of a mixture having 0% WFS was 2.02 MPa at 7 days of age. This strength increased by 3.34%, 6.89%, 9.25%, and 1.52% for mixtures incorporating 10%, 20%, 30%, and 40% WFS, respectively. After 28 days, for concrete mixtures comprising 10%, 20%, 30%, and 40% WFS, respectively, the STS was increased by 3.38%, 7.46%, 9.87%, and 2.50%, respectively. The strength was very analogous to that of the referenced mixture at the replacement stage of 40%.

At 56 days of age, concrete mixtures comprised of 10%, 20%, 30%, and 40% WFS achieved an increase of 5.48%, 11.17%, 13.14%, and 4.80%, respectively, over the STS of control concrete. At the age of 91 days, increments of 6.18%, 9.09%, 13.87%, and 16.07% were observed for mixtures comprising 10%, 20%, 30%, and 40% of WFS, respectively compared to the control concrete mixture. A maximum increase in STS for a concrete mixture containing 30% WFS was observed. As observed, the STS of WFS concrete mixtures increased with the age and with the WFS content up to a 30% level of substitution.

The change in STS with the content of WFS was similar to that observed for CS. The results of the present study are in good agreement with the findings of [4,5,8,9,10,11,12]. In this current investigation, the connection among CS and STS was observed in accordance with that of conventional concrete, i.e., the proportion of STS to CS for all the mixes was noted to be lying within the range of 8–15% [58]. Most of the concrete features are directly associated with its CS. Knowing its CS, the quality of concrete can be readily assessed. The findings show that the splitting tensile performance of plain concete with a partial substitution of natural sand by mass up to a substitution point of 30% is not adversely affected. Furthermore, the findings suggest a confirmed adverse effect on STS of plain concrete with the incorporation of WFS in excess of 40%.

The splitting tensile strength (STS) tests results of different mixtures consisting of WFS at various ages are shown in Figure 5. Concrete mixtures produced with WFS could be noted to expose greater STS than control concrete. The control mixture STS was around 3.28 MPa after 28 days of curing, and its value is very close to the mixture containing 40% WFS. The maximum strength was observed at a 30% replacement level at all the ages, as clear from Figure 5.

### 4.4. Flexural Strength Tests Results

Concrete tensile strength in terms of flexural strength (FS) is quite essential. The FS for concrete incorporating WFS as a partial substitute of sand was measured after the completion of 7 days, 28 days, 56 days, and 91 days of standard curing in accordance with ASTM C293 [49]. The results are shown in Figure 3. Similar to the CS and STS, with the incorporation of WFS as a partial sand substitution, the flexural strength (FS) has also been observed for the increments, and follows the same trend. At seven days of age, the FS was 5.02, 5.17, 5.37, 5.53, and 5.11 MPa, respectively for M-1, M-2, M-3, M-4, and M-5. For M-2, M-3, M-4, and M-5 mixtures, a marginal increment in FS over that of the control mixture was 2.99%, 6.82%, 10.00%, and 1.78%, respectively at 7 days of age. The FS of the control mixture M-1 (0% WFS) was 6.15 MPa, at 28 days, while mixtures of 10%, 20%, 30%, and 40% of WFS achieved FS of 6.34, 6.65, 6.78, and 6.27 MPa, revealing a marginal increment of 3.12%, 8.20%, 10.35%, and 2.01%, respectively compared to the control mixture. At 56 days of age, concrete mixtures of 10%, 20%, 30%, and 40% of WFS achieved an increase of 6.59%, 10.79%, 11.64%, and 4.14% respectively over the FS of control concrete. At 91 days of age, increments of 8.33%, 12.47%, 13.45%, and 4.14% were observed respectively, for mixtures consisting of 10%, 20%, 30%, and 40% WFS compared to the control concrete mixture. A marginal increase was observed for a substitution level of 30% at all the days of testing. A strength reduction was noted beyond a 30% replacemnt level. The FS value of M-1 and M-5 is very similar. This finding is in line with some other research findings such as those of [4,5,8,9,10,11,12].

**Figure 6:**
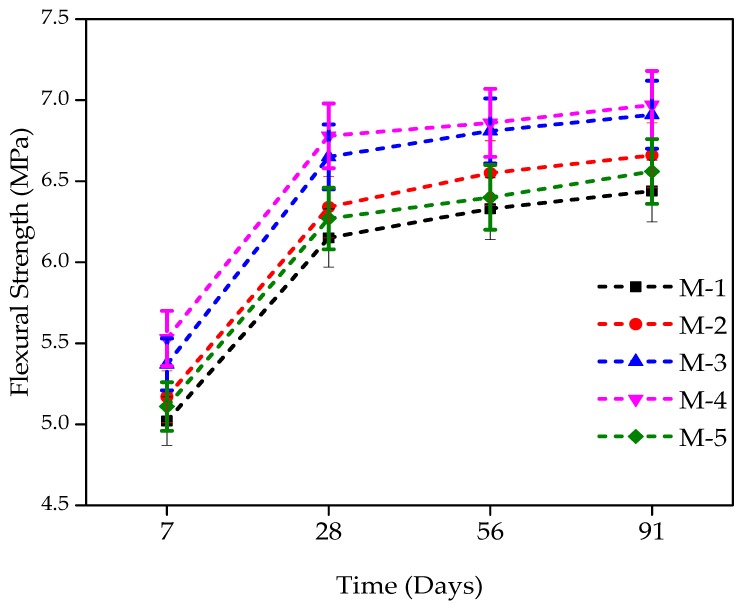
Flexural strength development of WFS concrete at different days [49].

The flexural strength (FS) tests findings of different mixtures incorporating WFS at various ages are shown in Figure 6. The FS possesses the same pattern as those of the STS and CS. Concrete mixtures produced with WFS could be noted as showing greater FS than control concrete. The control mixture’s FS was around 6.15 MPa after 28 days of curing, and its value was very close to the mixture containing 40% WFS. The maximum strength was observed at the 30% replacement level at all ages, as clear from Figure 6.

### 4.5. Variance in Batch Results

This section provides information about the variation in test results at all ages. It is important to mention here that the variance in the test results of different samples is within the limits prescribed by ASTM standards. As a case study, the variance in the results of one batch i.e., M-1, is specified and given in Table 6. All the values shown in Table 6 are within the limits of the ASTM standards specified for different tests [47,48,49].

### 4.6. Residual Compressive Strength

The concrete cylindrical specimens have been heated to 300, 400, 500, 600, 700, 800, 900, and 1000 °C. The specimens were put in an open-sky environment for 30 days before assessing the compressive strength. After exposure to elevated temperatures, the concrete cylinders were then evaluated for their compressive strength. The residual compressive strength (RCS) after fire exposure was calculated as the percentage of the respective unfired/unheated specimen’s compressive strength.

**Figure 7:**
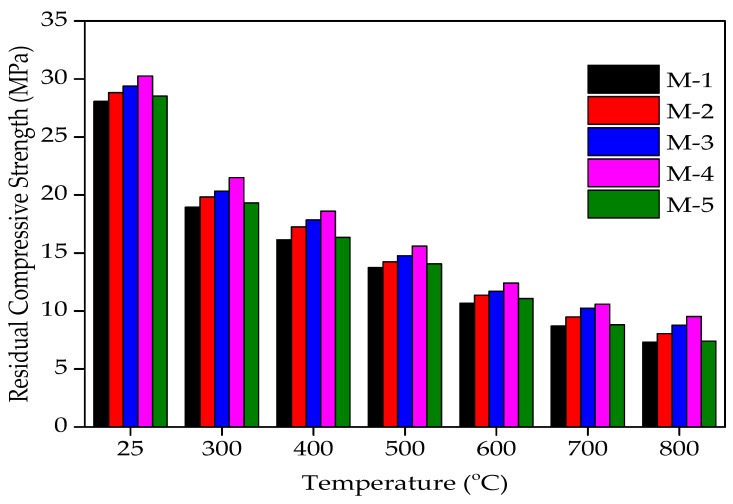
Residual compressive strength (RCS) of WFS concrete at various temperature levels.

**Figure 8:**
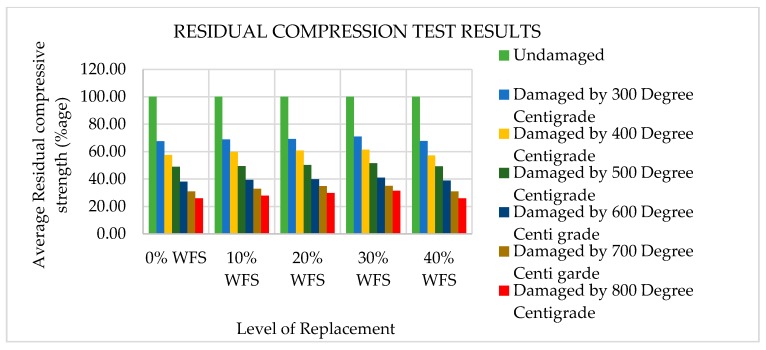
Variation in compressive strength after exposure to elevated temperatures.

All the specimens were tested for RCS at ambient temperature in this experimental work after being exposed to a one-month cooling period in an open-sky environment. At the moment of heating/cooling, no superimposed compressive load was applied. Since the maximum strength reduction occurs in unstressed concrete rather than stressed concrete at higher temperatures [59], It is more favorable to evaluate the RCS unstressed condition [59,60,61]. It can be seen from Figure 7 and Figure 8 that the RCS decreases mildly to a temperature level of 300 °C. Beyond 300 °C, it was observed that the reduction was very sharp because above 450 °C, the Ca_2_O_4_Si starts to degrade into CaO and SiO_2_. This is a permanent decomposition that results in a higher loss of strength [62]. The RCS after 30 days of cooling in surrounding air when exposed to 300 °C was found to be 67.50%, 68.83%, 69.17%, 71%, and 67.72% of unheated compressive strength values of 0%, 10%, 20%, 30%, and 40% of WFS concrete mixtures, respectively. The RCS after 30 days of cooling in surrounding air when exposed to 400 °C was found to be 57.50%, 59.86%, 60.71%, 61.50%, and 57.26% of the unheated compressive strength values for 0%, 10%, 20%, 30%, and 40% replacement of WFS, respectively. The RCS after 30 days of cooling in surrounding air when exposed to 500 °C was found to be 49.00%, 49.38%, 50.26%, 51.50%, and 49.30% of unheated compressive strength values for concrete mixtures consisting of 0%, 10%, 20%, 30%, and 40% WFS, respectively. The RCS after 30 days of cooling in surrounding air when exposed to 600 °C was found to be 38.00%, 39.41%, 39.81%, 41.00%, and 38.84% of the unheated compressive strength values for 0%, 10%, 20%, 30%, and 40% replacement of WFS, respectively. The RCS after 30 days of cooling in surrounding air when exposed to 700 °C was found to be 31.00%, 32.92%, 34.84%, 35.00%, and 30.87% of unheated compressive strength values for 0%, 10%, 20%, 30%, and 40% replacement of WFS, respectively. The RCS after 30 days of cooling in surrounding air when exposed to 800 °C was found to be 26.00%, 27.93%, 29.86%, 31.50%, and 25.89% of unheated compressive strength values for 0%, 10%, 20%, 30%, and 40% replacement of WFS, respectively. Strength loss was observed because after the maximum exposure to air (about one month), the CaO absorbed wetness and changed to Ca(OH)_2_ follow-on in additional cracking and a drop in the concrete’s RCS [63,64,65]. Maximum RCS was noted for the mixture containing 30% WFS as a fractional substitute of natural sand.

The residual compressive strength (RCS) of various concrete mixtures incorporating WFS at various elevated temperature levels are shown in Figure 7 and Figure 8. Strength reduction was observed from 300 °C temperature level for all of the mixtures. The maximum RCS at all of the temperature levels was noted for M-4 (30% replacement).

### 4.7. Ultrasonic Pulse Velocity Tests Results

**Figure 9:**
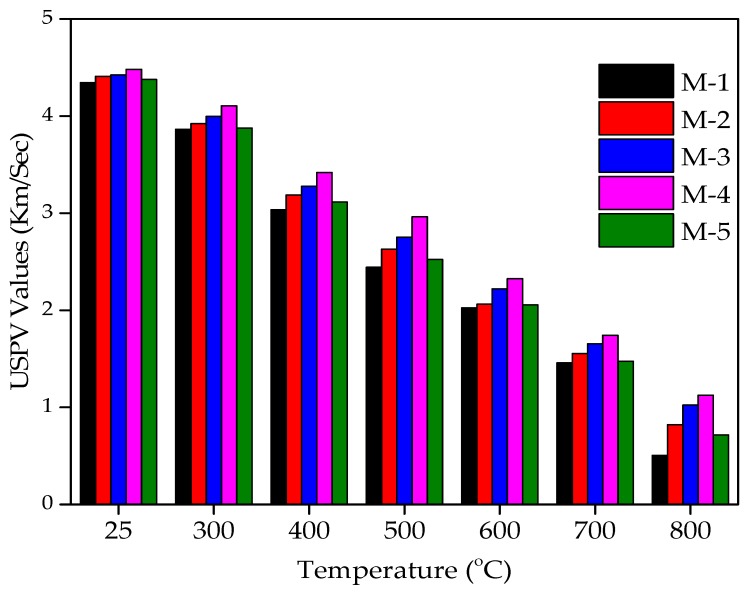
Ultrasonic pulse velocity (USPV) values of WFS concrete at various temperature levels.

**Figure 10:**
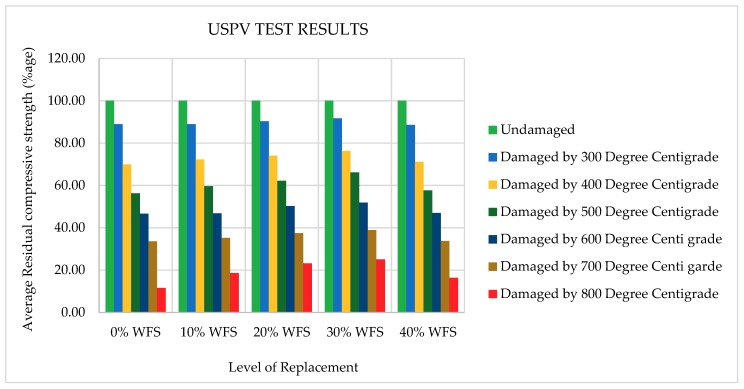
Variation in USPV values at elevated temperature levels.

The USPV values of various concrete mixtures incorporating WFS as a partial substitute of sand after being exposed to various levels of elevated temperatures are shown in Figure 9 and Figure 10. The USPV velocity reduction pattern started from the 300 °C temperature level for all the mixtures. M-4 had the maximum USPV values at all the temperature levels (30% replacement).

The USPV tests results are shown in Figure 9 and Figure 10. It can be observed from Figure 9 and Figure 10 that the USPV values increased with the increase in WFS content in concrete mixtures up to a substitution level of 30%. Each data point in Figure 9 and Figure 10 represents the average of three samples and four values measured for each specimen of ultrasonic pulse velocities. The USPV test was conducted on concrete specimens consisting of 0%, 10%, 20%, 30%, and 40% of WFS at the seventh day after 28 days of curing at ambient temperature (25 °C). The USPV test values were 4.345, 4.410, 4.425, 4.482, and 4.378 km/s for concrete mixtures consisting of 0%, 10%, 20%, 30%, and 40% WFS, respectively. The USPV outcomes are in good agreement with the compressive strength results. The USPV values after 30 days of cooling in surrounding air after exposure to 300 °C were found to be 3.864, 3.922, 3.997, 4.107, and 3.877 km/s for 0%, 10%, 20%, 30%, and 40% replacement levels of WFS, respectively. The USPV values after 30 days of cooling in surrounding air after exposure to 400 °C were found to be 3.036, 3.188, 3.277, 3.419, and 3.115 km/s for concrete mixtures consists of 0%, 10%, 20%, 30%, and 40% WFS, respectively. The USPV values after 30 days of cooling in surrounding air after exposure to 500 °C were found to be 2.443, 2.630, 2.752, 2.962, and 2.522 km/s for 0%, 10%, 20%, 30%, and 40% replacement levels of WFS, respectively. The USPV values after 30 days of cooling in surrounding air after experience to 600 °C were found to be 2.025, 2.063, 2.222, 2.324, and 2.055 km/s for 0%, 10%, 20%, 30%, and 40% replacement levels of WFS, respectively. The USPV values after 30 days of cooling in surrounding air after exposure to 700 °C were found to be 1.458, 1.554, 1.655, 1.742, and 1.475 km/s for concrete mixtures consists of 0%, 10%, 20%, 30%, and 40% WFS, respectively. The USPV values after 30 days of cooling in surrounding air after exposure to 800 °C were found to be 0.505, 0.821, 1.024, 1.125, and 0.716 km/s for 0%, 10%, 20%, 30%, and 40% replacement levels of WFS, respectively. It can be seen from the very low pulse velocities of Figure 5 and Figure 6 that the USPV of heated/fire-exposed concrete specimens decreases distinctly with the increasing level of exposure to heat/fire, and the drop in pulse values are prominently above the heated temperature level of 500 °C. It is very clear that the pulse velocities are very low above the level of 600 °C. This is because of the progress of wide-ranging cracks in the heated/fire-exposed concrete, which results in stopping the proceeding of the USPV, which caused low values of velocity [66,67]. It is obvious that the tendency of falling USPV values is similar to the tendency of falling levels of the concrete’s RCS. From the figure below, variation in USPV enhancement can also be easily observed. It was noted that the concrete mixture comprising of 30% WFS as a partial sand substitute exhibited higher USPV than that of the concrete control mixture.

### 4.8. Rebound Hammer Number (RHN) Tests Results

**Figure 11:**
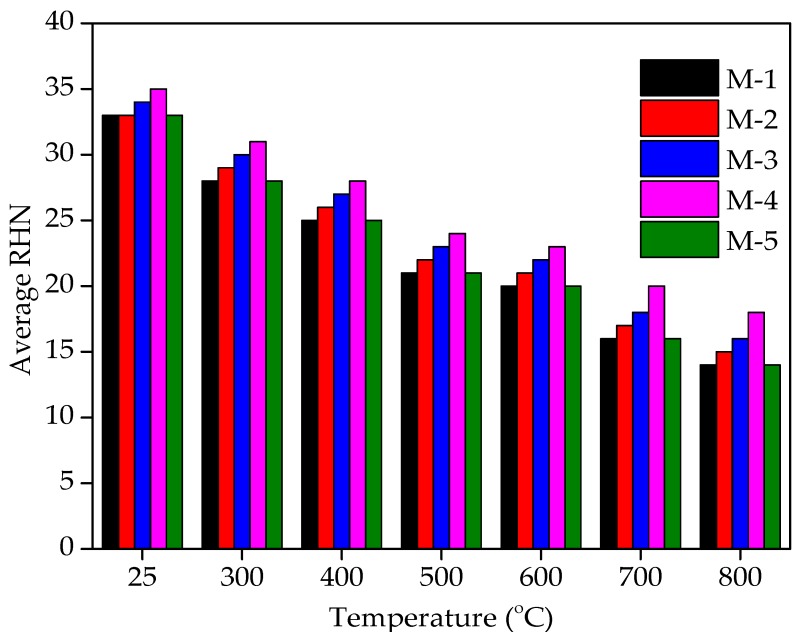
RHN values of WFS concrete at different temperature levels.

**Figure 12:**
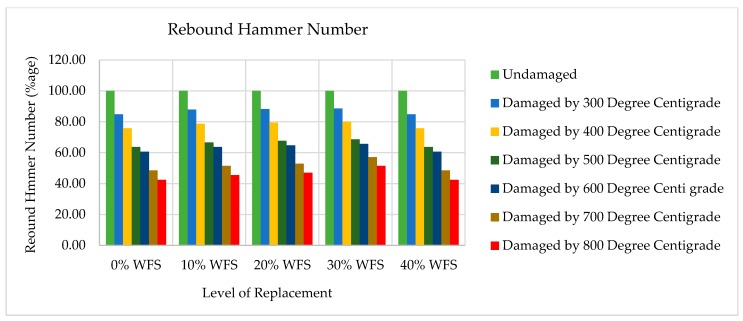
Variation in rebound hammer number (RHN) with elevated temperatures.

The RHN tests results are shown in Figure 11 and Figure 12. With the increase in WFS content in concrete mixtures, it can noted that RHN values improved. Each data point in Figure 11 and Figure 12 represents the average of three samples and six values measured for each specimen of rebound hammer number. The RHN test was conducted on concrete specimens consisting of 0%, 10%, 20%, 30%, and 40% of WFS on the seventh day after 28 days of curing at ambient temperature (25 °C). The RHN test values are 33, 33, 34, 35, and 33 for concrete mixtures consists of 0%, 10%, 20%, 30%, and 40% WFS, respectively at ambient temperature. The RHN results are in good agreement with the compressive strength and USPV results. The RHN values after 30 days of cooling in surrounding air after experience to 300 °C were found to be 28, 29, 30, 31, and 28 for 0%, 10%, 20%, 30%, and 40% replacement levels of WFS, respectively. The RHN values after 30 days of cooling in surrounding air after exposure to 400 °C were found to be 25, 26, 27, 28, and 25 for for concrete mixtures consists of 0%, 10%, 20%, 30%, and 40% WFS, respectively.

The RHN values after 30 days of cooling in surrounding air after exposure to 500 °C were found to be 21, 22, 23, 24, and 25 for 0%, 10%, 20%, 30%, and 40% replacement levels of WFS, respectively. The RHN values after 30 days of cooling in surrounding air after experience to 600 °C were found to be 20, 21, 22, 23, and 21 for 0%, 10%, 20%, 30% and 40% replacement levels of WFS, respectively. The RHN values after 30 days of cooling in surrounding air after experience to 700 °C were found to be 16, 17, 18, 20 and 16 for for concrete mixtures consists of 0%, 10%, 20%, 30%, and 40% WFS, respectively. The RHN values after 30 days of cooling in surrounding air after exposure to 800 °C were found to be 14, 15, 16, 18 and 14 for 0%, 10%, 20%, 30%, and 40% replacement levels of WFS, respectively. It can be seen from the very low rebound hammer number of Figure 7 and Figure 8 that the RHN of heated/fire-exposed concrete specimens decreases distinctly with the increasing level of exposure to heat/fire, and the drop in RHN values is prominently above the level of heated temperatures of 500 °C. It is very clear that RHN values are very low above the level of 600 °C. It is obvious that the tendency of falling in RHN values is similar to the tendency for the RCS of concrete and USPV values to drop. It was observed that concrete mixture consisting of 30% WFS as a partial sand replacement showed higher RHN than control concrete mixture.

### 4.9. Relationship between the Compressive Strength and USPV

**Figure 13:**
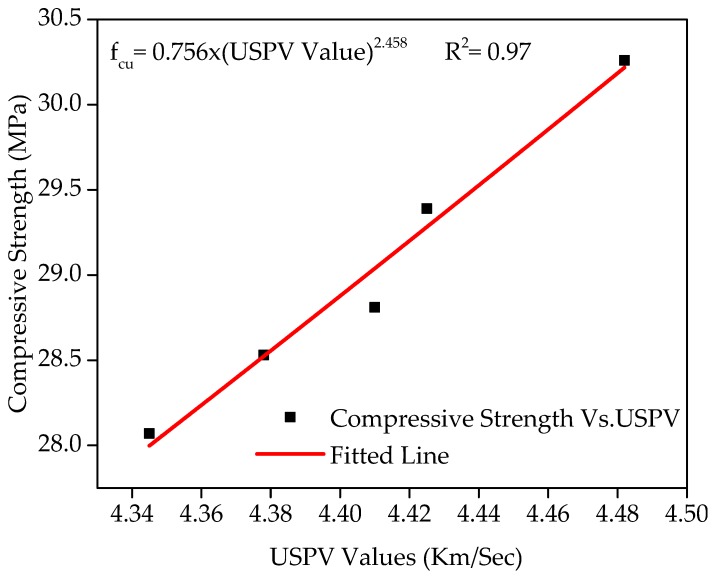
Relationship of compressive strength versus USPV.

Figure 13 displays the connection between compressive strength and the USPV test. As the value of R^2^ exceeds 0.94, an association in the form of {0.756 × (USPV value)^2.458^, R^2^ = 0.97} seems to be fit the data connection. A higher value of coefficient of determination shows that the CS has a strong connection with the USPV test. In general, an increase in the compressive strength with the inclusion of WFS increases the value of the USPV test, which is the indication of the concrete’s quality and homogeneity.

### 4.10. Relationship between the Compressive under Different Temperature and WFS Content

**Figure 14:**
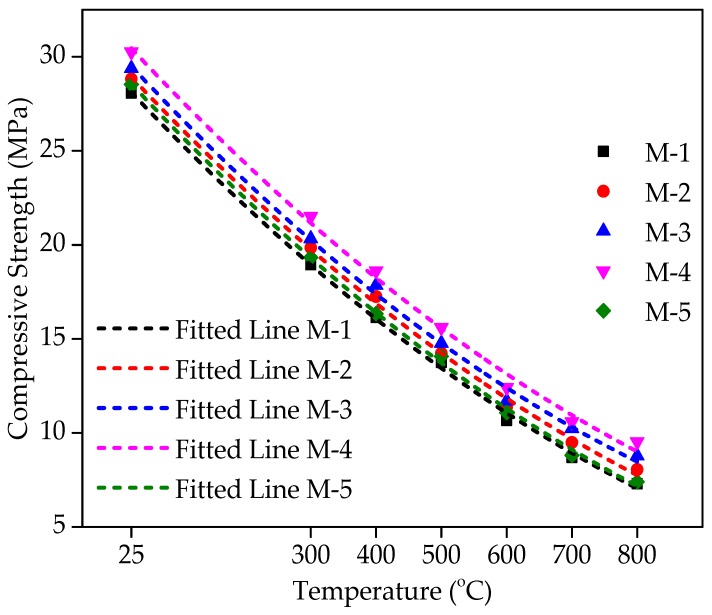
Relationship between the compressive under different temperatures and WFS content.

Figure 14 displays the connection between compressive strength under various levels of temperature and WFS content. Compressive strengths of various mixtures incorporating WFS as a partial substitute of sand at ambient temperature and elevated temperatures have been shown in Figure 14. Figure 14 demonstrates very obviously that M-4 has a peak value of compressive strength (CS) at ambient temperature and residual compressive strength (RCS) after exposure to elevated temperatures. It can be seen that the RCS decreases insignificantly to a temperature level of 300 °C. Beyond 300 °C, it was observed that the reduction was very sharp, because above 450 °C, the Ca_2_O_4_Si starts to degrade into CaO and SiO_2_. This is a permanent decomposition that results in a higher loss of strength [62]. Maximum strength loss was observed because upon the maximum exposure to air (at about one month), the CaO absorbed wetness and changed to Ca(OH)_2_ follow-on in additional cracking and a drop in the concrete’s RCS [63,64,65].

As the value of R^2^ exceeds 0.94, a polynomial association in different forms seems to be the best data connection with the various values of temperature and WFS contents. A coefficient of determination of R^2^ = 0.99 shows a strong data connection. A higher coefficient of determination value shows that compressive strength has a strong connection with various WFS content and temperature levels. Equations for each level of replacement and temperature are given below in Equations (1)–(5).

(1) $$\begin{array}{ccc} f_{\mathrm{cuM}-1}=29.04-0.0374\times T+1.2448\times 10^{-5}\times T^{2} & {25\;}^{\circ }C\leq T\leq {800\;}^{\circ }C; & R^{2} \end{array}=0.99$$

(2) $$\begin{array}{ccc} f_{\mathrm{cuM}-2}=29.84-0.0372\times T+1.988\times 10^{-5}\times T^{2} & {25\;}^{\circ }C\leq T\leq {800\;}^{\circ }C; & R^{2} \end{array}=0.99$$

(3) $$\begin{array}{ccc} f_{\mathrm{cuM}-3}=30.44-0.0379\times T+1.3403\times 10^{-5}\times T^{2} & {25\;}^{\circ }C\leq T\leq {800\;}^{\circ }C; & R^{2} \end{array}=0.99$$

(4) $$\begin{array}{ccc} f_{\mathrm{cuM}-4}=31.38-0.0377\times T+1.2222\times 10^{-5}\times T^{2} & {25\;}^{\circ }C\leq T\leq {800\;}^{\circ }C; & R^{2} \end{array}=0.99$$

(5) $$\begin{array}{ccc} f_{\mathrm{cuM}-5}=29.491-0.0376\times T+1.2148\times 10^{-5}\times T^{2} & {25\;}^{\circ }C\leq T\leq {800\;}^{\circ }C; & R^{2} \end{array}=0.99$$

### 4.11. Relationship between the RHN under different Temperatures and WFS content

**Figure 15:**
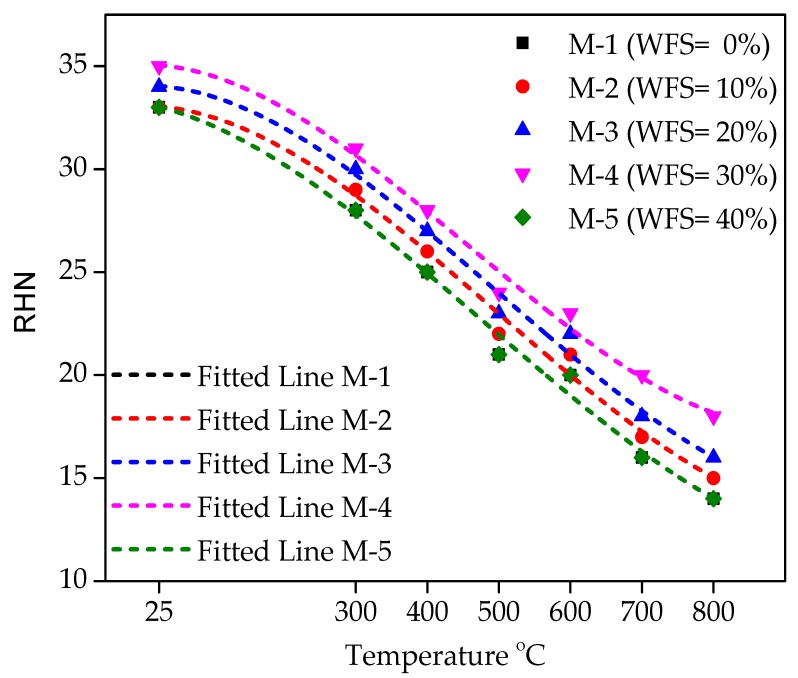
Relationship between the RHN under different temperature levels and WFS content.

Figure 15 displays the connection between the RHN test under various levels of temperature and WFS content. The RHN test of various mixtures incorporating WFS as a partial substitute of sand at ambient temperature and elevated temperatures is shown in Figure 15. The pattern of increasing/decreasing RHN values and compressive strength at varying temperatures and WFS content is very evident. At ambient temperature and after exposure to higher temperatures, mixture M-4 has a peak value of RHN at all the temperature levels. It is also noticeable that the RHN values are reduced with the increasing temperature levels, which indicates that the concrete quality is structurally influenced by higher temperatures. The RHN values for M-1 and M-5 concrete mixtures are very similar to each other at all the levels of elevated temperatures.

It can be seen that the RHN decreases inconsequentially to a temperature level of 300 °C. Beyond 300 °C, it was observed that the reduction was very sharp because above 450 °C, there was a breakage of CSH and some volumetric conversions within the structure of concrete. In addition, crakes and void generation occurs due to elevated temperature damage in the concrete matrix, which leads to a decrease in RHN values.

As the value of R^2^ exceeds 0.94, a polynomial association in different forms seems to be the best data connection with the various values of temperature and WFS contents. A coefficient of determination of R^2^ = 0.99 shows a strong data connection. A higher value of coefficient of determination shows that the RHN value has a strong connection with various WFS content and temperature levels. Equations for each level of replacement and temperature are given below (Equations (6)–(10)).

(6) $$\begin{array}{c} RHN_{M-1}=33.232-(0.00679\times T)-4.815\times 10^{-5}\times {\left(T\right)}^{2}+3.326\times 10^{-8}{\left(T\right)}^{3}\\ {25\;}^{\circ }C\leq T\leq {800\;}^{\circ }C;\;R^{2}=0.99 \end{array}$$

(7) $$\begin{array}{c} RHN_{M-2}=33.070-(1.981\times 10^{-4}\times T)-6.1269\times 10^{-5}\times {\left(T\right)}^{2}+4.103\times 10^{-8}{\left(T\right)}^{3}\\ {25\;}^{\circ }C\leq T\leq {800\;}^{\circ }C;\;R^{2}=0.99 \end{array}$$

(8) $$\begin{array}{c} RHN_{M-3}=34.070-(1.981\times 10^{-4}\times T)-6.1269\times 10^{-5}\times {\left(T\right)}^{2}+4.103\times 10^{-8}{\left(T\right)}^{3}\\ {25\;}^{\circ }C\leq T\leq {800\;}^{\circ }C;\;R^{2}=0.99 \end{array}$$

(9) $$\begin{array}{c} RHN_{M-4}=35.082-(2.895\times 10^{-4}\times T)-6.398\times 10^{-5}\times {\left(T\right)}^{2}+4.648\times 10^{-8}{\left(T\right)}^{3}\\ {25\;}^{\circ }C\leq T\leq {800\;}^{\circ }C;\;R^{2}=0.99 \end{array}$$

(10) $$\begin{array}{c} RHN_{M-5}=33.232-(0.00679\times T)-4.815\times 10^{-5}\times {\left(T\right)}^{2}+3.326\times 10^{-8}{\left(T\right)}^{3}\\ {25\;}^{\circ }C\leq T\leq {800\;}^{\circ }C;\;R^{2}=0.99 \end{array}$$

### 4.12. Spalling and Cracking Behavior of Foundry Sand Concrete

Concrete spalling is a major parameter when concrete is to be exposed to elevated temperatures. Due to the significant loss of concrete strength at elevated temperatures, explosive spalling can cause a complete or abrupt failure. The temperature range is in between 300–650 °C. Many factors were identified as affecting explosive spalling. These factors including the age, moisture content, type of gravel and sand used, curing method, and rate of heating. Low heating rates reduced the risk of spalling.

It is considered that pore pressure stresses play a major contribution in explosive spalling. Due to low tensile strength, chances of explosive spalling could be more in normal strength concrete [31]. In this present study, explosive spalling was observed at 650 °C and 730 °C for concrete mixtures containing 0% waste foundry sand and 10% waste foundry sand, respectively. No explosive spalling was observed for other concrete mixtures. Maximum surface spalling was observed at 900 °C and 1000 °C, along with extensive cracks, and the specimens were not able even to perform a single test. No indication of any observable cracks was found in the concrete cylinders subjected to 300 °C to 500 °C. Although minor cracks were observed when concrete was subjected to 600 °C. At 700 °C, significant observable cracks were seen on the surface of all specimens.

## 5. Conclusions

In this study, concrete mixtures were prepared by the inclusion of WFS as a partial replacement of natural/regular sand, and then its effect was investigated on the fresh properties i.e., slump and compacting factor tests and strength properties i.e., compressive strength, splitting tensile strength, and flexural strength at the age of 7, 28, 56, and 91 days. The effect of inclusion of waste foundry sand as a partial replacement of sand was also investigated in terms of the effect of elevated temperatures ranging from 300 to 800 °C at the increment of 100 °C on the compressive strength of foundry sand concrete. The USPV and rebound hammer tests were performed at the age of 28 days before exposure to elevated temperatures and on the 30th day after being exposed to elevated temperatures. The spalling phenomenon in foundry sand concrete and the control samples was carefully observed in this investigation. The findings are summarized as follows.

1. The fresh properties for all replacements were observed to be almost similar with the control mixture up to a 30% replacement level. The compacting factor values and slump values were decreased with the inclusion of waste foundry sand. Up to 30%, no severe effect on the slump and compacting factor tests results were observed.
2. The strength properties i.e., compressive strengths, splitting tensile strengths, and flexural strengths values of foundry sand concrete mixtures at all ages were found to be higher than those of the control mixtures. The maximum values for strength properties were observed at a 30% replacement level. The substitutional level of normal sand with WFS was found to be optimum at 30%, and should not exceed 40%. The inclusion of waste foundry sand increases the rebound number and USPV values, which is the indication of the quality, homogeneity, and impermeableness of foundry sand concrete.
3. The residual compressive strength of concrete decreases with increasing temperature. Air curing results in a further reduction of residual compressive strength. The residual compressive strength of the concrete containing waste foundry sand as a partial replacement of natural sand on the 30th day of air/open-sky cooling at ambient temperature after exposure to 300, 400, 500, 600, 700 and 800 °C was reduced in the range of 29.00% to 32.5%, 38.5% to 42.5%, 48.5% to 51.00%, 59.00% to 62.00%, 65.00% to 69.00%, and 68.5% to 74% of unheated compressive strength values for 0% to 40% replacement of WFS, respectively.
4. The pattern of increase in USPV, rebound hammer, compressive strengths tests at ambient temperature before exposure to elevated temperatures, and the pattern of reduction in USPV, rebound hammer, and residual compressive strength test of foundry sand concrete was found to be similar with the increasing temperature.
5. The maximum value for all the tests was observed before and after exposure to elevated temperatures for a 30% replacement level of waste foundry sand.

Based on the above findings, it can be concluded that waste foundry is a suitable candidate for the partial replacement of natural sand up to 30% in making concrete.

## Figures and Tables

**Table 1:** Chemical composition (CC) and physical properties of Portland cement.

Chemical Composition of Cement		Physical Properties of Cement	
Components	Content (%)	Parameter	Value
CaO	63.47	Specific Surface	322 m^2^/kg
SiO_2_	22.00	Consistency	29%
Al_2_O_3_	5.50	Initial Setting Time	1 h and 42 min
MgO	1.70	Final Setting Time	3 h and 55 min
SO_3_	1.82	Specific Gravity	3.05
Fe_2_O_3_	3.50	-	-
Na_2_O	0.20	-	-
K_2_O	1.00	-	-
Loss of Ignition (LOI)	0.64	-	-

**Table 2:** Chemical composition (CC) of waste foundry sand.

Component	Contents (%)	Requirements as per American Foundry Men’s Society, 1991
SiO_2_	88.50	87.9%
Al_2_O_3_	4.63	4.70%
Fe_2_O_3_	0.83	0.94%
MgO	0.21	0.30%
CaO	0.90	0.14% (Min.)
Na_2_O	0.02	–
K_2_O	0.01	–
Sulphates	0.03	0.09%
Loss of Ignition (LOI)	4.37	5.15% (max.)

**Table 3:** Physical properties of aggregates.

Property	Natural Sand	Coarse Aggregate	Waste Foundry Sand
Specific Gravity	2.61	2.66	2.55
Unit Weight (kg/m^3^)	1720	1600	1555
Fineness Modulus	2.60	-	1.90
Water Absorption (%)	0.67	0.73	1.48

**Table 4:** Mix proportions containing WFS.

Mix Designation	Level of Replacement	Cement (kg/m^3^)	WFS (kg/m^3^)	Fine Aggregate (kg/m^3^)	Coarse Aggregate (kg/m^3^)	Water (kg/m^3^)
M-1	0%	436.4	0	654.5	1309.1	187.6
M-2	10%	436.4	65.4	589.1	1309.1	187.6
M-3	20%	436.4	130.9	523.6	1309.1	187.6
M-4	30%	436.4	196.4	458.2	1309.1	187.6
M-5	40%	436.4	261.8	392.7	1309.1	187.6

**Table 5:** Slump and compacting factor (CF) values at different substitution levels.

Mix Designation	Level of Replacement	Actual Slump Achieved (mm)	Slump Value (%age reduction)	Compacting Factor Value
M-1	0%	32	Reference Slump	0.85
M-2	10%	30	6.25	0.84
M-3	20%	30	6.25	0.84
M-4	30%	27	15.62	0.83
M-5	40%	22	31.25	0.81

**Table 6:** Variance of results in one batch (M-1).

Testing Period	No. of Samples	Compressive Strength (MPa)	Variance (%)	Splitting Tensile Strength (MPa)	Variance (%)	Flexural Strength (MPa)	Variance (%)
7 Days	Sample 1	20.81	0.00%	1.84	0%	4.89	0%
	Sample 2	21.80	4.76%	2.00	9%	5.32	9%
	Sample 3	22.35	7.44%	2.05	12%	5.41	11%
28 Days	Sample 1	27.16	0.00%	3.02	0%	5.99	0%
	Sample 2	28.09	3.42%	3.36	11%	6.14	2%
	Sample 3	28.96	6.63%	3.43	13%	6.31	5%
56 Days	Sample 1	29.36	0.00%	3.31	0%	6.04	0%
	Sample 2	29.56	0.68%	3.39	2%	6.37	5%
	Sample 3	29.96	2.04%	3.50	6%	6.57	9%
91 Days	Sample 1	30.25	0.00%	3.34	0%	6.18	0%
	Sample 2	30.57	1.06%	3.44	3%	6.57	6%
	Sample 3	30.80	1.82%	3.68	10%	6.58	7%
